# Teachers’ Conceptions of Teaching Chinese Descriptive Composition With Interactive Spherical Video-Based Virtual Reality

**DOI:** 10.3389/fpsyg.2021.591708

**Published:** 2021-02-03

**Authors:** Mengyuan Chen, Ching-Sing Chai, Morris Siu-Yung Jong, Michael Yi-Chao Jiang

**Affiliations:** Department of Curriculum and Instruction & Centre for Learning Sciences and Technologies, The Chinese University of Hong Kong, Shatin, China

**Keywords:** virtual reality, writing education, teacher conception, phenomenography, qualitative research method

## Abstract

Phenomenographic research about teachers’ conception of teaching has consistently revealed that teachers’ conception of teaching influence their classroom practices, which in turn shape students’ learning experiences. This paper reports teachers’ conceptions of teaching with regards to the use of interactive spherical video-based virtual reality (ISV-VR) in Chinese descriptive composition writing. Twenty-one secondary teachers in Hong Kong involved in an ISV-VR-supported Chinese descriptive writing program participated in this phenomenographic study. Analyses of the semi-structured interviews establish seven conception categories that are specifically related to the use of ISV-VR for descriptive Chinese composition writing: (1) offering students more observational opportunities; (2) improving students’ writing skills; (3) promoting students’ learning participation and motivation; (4) shifting learning from teacher-centric to student-centric, (5) enhancing collaborative learning among students; (6) cultivating students’ positive values and moral character, and (7) shaping students’ self-identity as “writers.” The concurrent and convenient access to the ISV-VR resources was for the teachers an enriched and supportive environment for them to cultivate students’ writer identity. In addition, it was discovered that the structural relationships of the conceptions may be better organized along three axes of continuum: conception’s orientation, teaching attention locus, and understanding of writing. These categories form a hierarchy from skill-oriented to community-oriented, and finally to identity-oriented conception. The findings may provide researchers and practitioners with novel insight into the teaching of composition writing in the contexts of L1 acquisition supported by virtual reality technology.

## Introduction

Literacy has always been a key competency and one of the key intellectual infrastructural elements that students need to develop (Earnshaw, 2007; Bazerman, 2009; MacArthur et al., 2016). Among the four language skills that constitute literacy, writing is recognized not only as a means of communication but also a means to improve reading skill, comprehension, and critical thinking (How and Larkin, 2013; Teng, 2016; Kong, 2018; Wang and Matsumura, 2019). Many language educators consider writing as the pinnacle of language education (Earnshaw, 2007; Bazerman, 2009; MacArthur et al., 2016).

In addition to the focus on writing, many contemporary language education curriculum emphasize the development of students’ creativity, critical thinking, and positive values and attitudes (Choi, 2016; Macalister and Nation, 2019; Mickan and Wallace, 2019). Similarly, the Chinese language education (CLE) curriculum guide in Hong Kong also highlights the importance of improving students’ communication, collaboration, self-learning skills, and culture learning supported by Information and Communication Technologies (ICT) (Curriculum Development Council of Hong Kong, 2017). These emphases can be broadly classified as 21st-century teaching and learning practices (Chai et al., 2019).

Despite the above emphases, the school’s daily writing exercises are centered on knowledge presentation (take notes, write summaries, complete worksheets, etc.) (Teale and Yokota, 2000). Students may not prefer such pedagogical practices that fail to engage them cognitively and socially with technological supports (Chai et al., 2019). In addition, such practices may not address the perpetual problem of students’ weakness in composition writing in general, and in descriptive writing in particular (Holliway, 2004; Akincilar, 2010; Carter, 2015). This study leverages on the interactive spherical video-based virtual reality (ISV-VR) technology to transform the writing pedagogy for descriptive writing. As teachers are the key agents in all education reform (Jong, 2019); and studies showed that teachers’ conceptions of writing instruction play a role in how they implement writing programs within their classrooms (Lin, 2016; Kong, 2018; Wang and Matsumura, 2019); this study aims to explore how teachers conceive and hence enact descriptive writing pedagogy employing the affordances of ISV-VR technology. This study contributes to the current literature by describing a range of conceptions of teaching that may empower or limit teachers’ use of virtual reality (VR) technology for the teaching of composition writing. Knowledge of teachers’ conceptions may also provide useful insight for teacher educators and policymakers to enhance educational practices (Kelly and Beth, 2017).

## Literature Review

### Challenges in Teaching Descriptive Composition

Among various genres of composition, essays involving substantial descriptions are usually among the assignments that students must complete in composition classes (Holliway, 2004; Akincilar, 2010; Carter, 2015). According to Babayigit and Stainthorp (2011), descriptive writing is a comprehensive process that relies on the integration of different levels of processing skills such as working memory and transcription-related abilities. Successful descriptive composition affords readers a vivid environment to situate the themes of the essay through the writer’s words. Experienced writers can create “pictures” with words (Holliway, 2004) but many novice writers fail to visualize the environment with words. Wilhelm (2008) stated that unless students “see” something in their mind, they will not be able to write about it. Researchers have reported that teaching secondary school students how to depict vivid and rich pictures for readers to fully understand the text is always a pedagogical challenge (e.g., Holliway, 2004; Akincilar, 2010; Babayigit and Stainthorp, 2011; Carter, 2015). This could be due to students’ lack of vocabulary or writing skills, but it could also be due to pedagogical gaps in teaching.

In addition to the general writing skills including the choice of vocabulary, sentence structure, grammar and mechanics (Nair and Sanai, 2018; Chen et al., 2019), there are some descriptive techniques commonly used in Chinese landscape literature. The advocated descriptive writing techniques has been listed in the curriculum guide. According to Curriculum Guide for Chinese Language Education and Learning (Primary 1 to Secondary 6), the writing techniques of descriptive composition include the static descriptive method, which refers to a method of writing that creates vivid and concrete images of people and objects by describing them in a static state; and the dynamic description, which refers to a method of describing the moving state of people and objects to create concrete and lifelike images for the readers. In addition, the walking description method requires authors to observe scenes while walking, and describe the changing scenes; and the fixed-point description method is to choose an appropriate and fixed perspective to observe scenes, and to describe the object such as from top to bottom or left to right. Lastly, the sensory description method means that the author writes about people or scenes base on what he sees, hears, tastes, smells and touches (Curriculum Development Council of Hong Kong, 2017).

Despite the clear articulation of such techniques, a recent report from the high-stakes diploma of secondary education examination in Hong Kong shows that the lack of detailed descriptions, monotonous expressions, and unaffectionate descriptions are major problems with Hong Kong students’ Chinese compositions (Hong Kong Examination and Assessment Authority, 2018). The report attributed these problems as the result of students failing to make observations in daily life and the lack of an in-depth understanding of their communities. Similar problems in students’ performance in descriptive writing have been commonly reported (Holliway, 2004; Akincilar, 2010; Babayigit and Stainthorp, 2011; Carter, 2015; Huang et al., 2020). The challenges may lie in facilitating students’ understanding about how description are connected with other parts of the essay, and the socio-historical significance of the described environment. Teaching students to observe their environment sensitively so as to build personal affective connections with the communities seems to be a pedagogical gap for CLE. ISV-VR is a new educational tool that supports learning and teaching activities in which “students’ observation” is an important pedagogical component (e.g., Geng et al., 2019; Chien et al., 2020; Jong et al., 2020). It also greatly saves the cost and time of VR courseware development (Ozkeskin and Tunc, 2010; Mohiuddin et al., 2016; Repetto et al., 2018). Moreover, the technical skills of producing ISV-VR content is easy to master, thus allowing school teachers to develop immersive materials according to their own teaching needs (Chen et al., 2019; Chien et al., 2020). Given the affordances of ISV-VR that may address the pedagogical challenge of fostering descriptive writing, the potential of ISV-VR supported Chinese writing was introduced to the teachers and relevant training for the technical skills; and resources with possible lesson activities was provided for the teachers to engage students in immersive appreciation of various places in Hong Kong. This study looks into the teachers’ conceptions of designing and using ISV-VR to teach descriptive writing (for convenience, VRDW).

### Affordances of VR in Writing Instruction

Advancements in ICT have created the demand for innovative use of digital technology in the language education context (Lin, 2015; Kozar, 2016; Liu et al., 2017; Taghizadeh and Yourdshahi, 2019). VR technology is a potential tool to remedy the lack of opportunities for students to gather writing materials through detailed observation of phenomena. VR enables students to immerse themselves in a virtual environment comparable to the authentic world (Ozkeskin and Tunc, 2010; Lin and Lan, 2015; Mantziou et al., 2018; Repetto et al., 2018). It provides a safe and self-directed environment that enables students to observe places at length, with or without their teachers’ guidance in the classroom.

Literature from immersive language learning approaches highlights the need for a rich and authentic environment to provide the socio-cultural context for learners to develop nuanced understandings of language in use (Cummins, 2000; Marsh et al., 2000; Swain and Lapkin, 2005). Research in embodied cognitive science also attests that situated immersive learning can stimulate students’ learning motivation, enhance the learning experience, and encourage knowledge transfer (Marsh et al., 2000; Wilson, 2002; Swain and Lapkin, 2005; Steele et al., 2018). The content presented through interactive spherical video is more realistic than the 3D animation environment. The images employed in ISV-VR are filmed in the real world, and thus to present the complexity, diversity and details of the real world (Lin et al., 2019; Chang et al., 2020). Developers can also add a variety of human-computer interactions to the VR environment (Geng et al., 2019; Chien et al., 2020). The authors’ previous research results of VRDW indicated that the VRDW courses could improve students’ engagement and learning motivation in writing courses, and their writing performance regarding to the writing skills and themes of compositions (Chan et al., 2019; Chao et al., 2020). Findings from another study adopted spherical video-based VR (SVVR) to teach descriptive writing also showed that the SVVR writing approach could improve students’ writing performance in terms of content and appearance as well as their creativity tendency and writing self-efficacy, while also reducing their cognitive load (Huang et al., 2020).

Despite the aforementioned affordances, Lin and Lan’s (2015) review of the use of VR in language learning indicated that only 3 of the 29 studies they reviewed studied the teachers’ perceptions or awareness of VR. These studies were focused on second language and other language skills instead of writing. A recent search (dated 26th November 2020) using the most comprehensive database (i.e., the Google Scholar) with the search term “composition writing” AND “Chinese language” AND “virtual reality” yielded eight returns, none of which investigate how teachers’ conception or beliefs could influence how they conceive the role of VR for descriptive composition writing. It seems important for educational researcher to understand the conceptions that shape teachers use of the VR technology for writing curriculum because teachers’ conception of technology has been shown to influence their practices (Alshahrani and Al-Shehri, 2012; Lameras et al., 2012; Lin, 2015; Kozar, 2016; Hsieh and Tsai, 2017). The current study thus sought to explore teachers’ conceptions of VR usage in L1 instruction with a special focus on the teaching of writing.

### Teacher’s Conceptions of Writing Instruction

“Conceptions” were defined by Pratt (1992) as specific meanings associated with phenomena that mediate the conceiver’s reactions to situations involving these phenomena. In the context of educational research, conceptions of teaching and learning are studied employing the methods from phenomenography, which studies how people experience and understand phenomena in the world around us (Marton and Pong, 2005). Phenomenographic analysis methods can be used to study meaning, understanding, and conceptual variation. Rather than describing the real situation of the world, phenomenography focuses more on studying people’s experience from the perspective of the participants. The core of phenomenographic study is the concept of “essence.” For studying people’s experiences of a certain aspect of reality, the “essence” here refers to the common and internal subjective meanings of that aspect (Paakkari et al., 2011).

In the context of education, conceptions of teaching have been developed as a distinctive field of research (Thompson, 1992). The teaching conception refers to a coherent system of knowledge and beliefs about teaching and related phenomena (Vermunt and Vermetten, 2004). Through phenomenographic research, conceptions of writing instruction have been described as ranging along the continuum of behaviorist and constructivist orientation in some literature (e.g., Lipson et al., 2000; McCarthey and Mkhize, 2013; Newell et al., 2014). Teachers who hold behaviorist-orientated conceptions are likely to emphasize the teaching of individual writing skills (Lipson et al., 2000). Teachers with constructivist-orientated conceptions tend to cultivate students’ rhetorical style and voice (Lipson et al., 2000; McCarthey and Mkhize, 2013). Teachers’ conceptions can also form a spectrum and can be placed into a hierarchy ranging from superficial to sophisticated, teacher-focused to student-focused, skill-oriented to meaning-oriented, and product-focused to process-focused (e.g., Boulton-Lewis et al., 2001; Kong, 2018; Wang and Matsumura, 2019).

These studies provide us with a deeper understanding of language teachers’ conceptions of writing education. Recent studies indicate that language teachers conceive that using information and communication technology could foster students’ thinking skills and promote learning beyond school (Alshahrani and Al-Shehri, 2012; Liu et al., 2017). However, none of these studies focus on teachers’ conceptions of using VR to teach first language writing. These studies advocate that more nuanced studies are needed in this research field. The current study thus proposes two research questions: (1) what are the teachers’ conceptions of VRDW? (2) what are the variations in teachers’ conceptions of VRDW?

## Materials and Methods

In consideration of the students’ performance gaps and the current affordances of VR technology, this study launched a VRDW learning program for six Hong Kong secondary schools. The program aimed to offer students near real-life observation opportunities through ISV-VR with added pedagogical features to foster closer connections between the students and various Hong Kong communities. Communities in this study are defined as the places and the people living there.

### School Background and Participants

Twenty-one grade 7–9 secondary Chinese teachers from a school in Hong Kong participated in the current study. The school is well equipped with technologies and the leadership is enthusiastic about integrating ICT into the curriculum. The Chinese language department participated in the VRDW program. Teachers designed the teaching plan and the VR learning content, and implemented the teaching plan. All Chinese teachers who participated in the VRDW were approached and they agreed to participate. They have experienced adopting the VRDW for at least one design and implementation cycle. The background information of the participants is reported in Table 1. Seven of them are male (33%). The average age of participants is 32.3 (SD = 8.7). Six of them have a Master’s degree (29%) while the rest graduated as Bachelor (71%). Sixteen (76%) of them have a postgraduate diploma in education.

**TABLE 1:** Composition of the participants.

	Gender	Age	Chinese teaching seniority (year)	E-learning experience (year)	Degree	PGDE*
T1	Male	41–45	11–20	11–20	Bachelor	Y
T2	Female	20–25	1–3	1–3	Bachelor	Y
T3	Female	31–35	4–10	1–3	Bachelor	Y
T4	Female	41–45	11–20	<1	Master	Y
T5	Female	20–25	<1	<1	Bachelor	N
T6	Female	20–25	<1	<1	Bachelor	N
T7	Female	26–30	1–3	1–3	Bachelor	Y
T8	Female	41–45	11–20	<1	Master	Y
T9	Female	36–40	11–20	4–10	Bachelor	Y
T10	Male	26–30	<1	1–3	Bachelor	Y
T11	Female	20–25	<1	<1	Bachelor	Y
T12	Male	36–40	11–20	11–20	Master	Y
T13	Female	26–30	4–10	1–3	Bachelor	Y
T14	Male	20–25	<1	<1	Bachelor	N
T15	Female	20–25	<1	<1	Bachelor	Y
T16	Male	26–30	1–3	1–3	Master	N
T17	Male	26–30	1–3	1–3	Bachelor	Y
T18	Female	41–45	11–20	1–3	Bachelor	Y
T19	Female	46–50	11–20	1–3	Master	Y
T20	Female	26–30	4–10	1–3	Master	Y
T21	Male	41–45	11–20	4–10	Bachelor	N

*PGDE refers to Postgraduate Diploma in Education.

### Data Collection

The data in this study were collected through semi-structured interviews. The interviews were conversations around interview questions with the purpose of communicating and documenting the participant’s experience (Moustakas, 1994). The questions for interviews were adapted from previous research which explored teachers’ conceptions of adopting technology in teaching (Lameras et al., 2012; Hsieh and Tsai, 2017):

1. For what reason do you decide to use VR in teaching?
2. What is the role of VR in your teaching?
3. What are the differences between your VR writing class and your previous writing class?
4. Could you describe a VR writing lesson that you think is successful or unsuccessful?
5. Please give a formal definition of “VR supported descriptive writing learning.”

During the interviews, the interviewers asked the participants to give examples or more detailed explanations when the interviewers were not clear with the participants’ ideas (Creswell, 2003). The teachers were interviewed in Mandarin or Cantonese. The researchers also observed the classes before conducting the interviews in order to understand more fully and accurately about the teachers’ discourse, and made appropriate subtle adjustments to the interview questions. The participants were interviewed individually after they finished the VR curriculum implementation, and each interview lasted between 45–60 min. After the interviews, the audio recordings were transcribed verbatim.

### Data Analysis

This study adopted the phenomenographic approach to analyze the teacher interview transcripts (Marton and Pong, 2005). Phenomenology mainly includes two aspects: looking for variations in teachers’ conceptions and the structural relationship between these conceptions. In this study, we study teachers’ conceptions of VRDW from the two aspects. After transcription, the data were managed in Nvivo (Version 12) to facilitate the creation and manipulation of codes.

The transcripts were analyzed iteratively. The researchers first read the transcripts several times to familiarize themselves with the participants’ thoughts. Then used several keywords to mark the main meanings of teachers’ views on using ISV-VR to teach Chinese writing. Next, the researchers compared and contrasted the similarities and differences between the main meanings to form the structural relationship of teachers’ conceptions. Finally, based on the structural relationship, the researchers constructed a hierarchical structure of teachers’ conceptions of VRDW. It includes describing categories in more details, delineating categories based on dimensions, generating outcome space which shows the logical connections among categories of description (Marton and Pong, 2005). For example, the following passage was marked to belong to category 1 as “breaking time and space limitations” to summarize its main meaning.

“The important thing is that VR can break the constraints of time and space. For example, VR allows us to watch the night scene during the day, which is the advantage of this technology.”

For another two examples, we used “safe and effective way to replace field trips” to represent the main idea of the following two passages.

“In fact, using VR in writing class is more like an electronic version of field trips. Actually, I can’t take my whole class to have field trips often for safety and time problems. Such opportunities are very rare for students. VR can increase their opportunities for observation. So basically I think this is a ‘field trip in the classroom’.”

“Using VR can save a lot of time and is more convenient than traditional field trip.”

The following two passages was marked as “observe opportunities that hard to get in daily life” to summarize their main meanings.

“Students who use VR can look more carefully without causing misunderstanding and embarrassment, because many students will be embarrassed to observe in real life in crowded places, or observe other people.”

“There are things and sights in ISV-VR that I haven’t ever seen before. So, I think this is a good opportunity for students to observe things what they don’t have the chance to see in their daily life.”

Categories of the teachers’ conceptions of VRDW emerged through the comparison and contraction of these key meanings. These examples and some other passages all emphasized using ISV-VR as a way to let students have more chances to observe and experience the world out of the classroom. Thus, a category of “offering students more observational opportunities” was created.

Member checks were employed to improve the trustworthiness of this study. The researchers engaged the participants to review the researchers’ interpretation of their experiences and to suggest adjustments to better capture their perspectives. After forming the initial categories, we sought feedback from an 18 years veteran Chinese writing teacher and an educational researcher who have rich research experience in educational phenomenology and Chinese writing education for communicative validity check (Åkerlind, 2005). The categories were then improved and finalized based on the feedback.

## Results

Seven categories of teachers’ conceptions of teaching descriptive writing with ISV-VR generated through the analysis. The results show that the participating teachers viewed VRDW as a hierarchy of conceptions that includes (1) offering students more observation opportunities, (2) improving students’ writing skills, (3) promoting students’ learning participation and motivation, (4) shifting learning from teacher-centric to student-centric, (5) enhancing collaborative learning among students, (6) cultivating students’ positive values and moral character, and (7) shaping students’ self-identity as “writers.” The structural relationship of teachers’ conceptions are illustrated through three key aspects: (1) conceptions’ orientation, (2) teaching attention locus, and (3) understanding of writing. In the following sections, we first describe the seven categories in detail, followed by their structural relationship and hierarchy, and finally, present the distribution of the categories.

### Description of Categories

#### Offering Students More Observational Opportunities

In this category, ISV-VR is described as a tool that affords the means for students to have more observational opportunities. Teachers view using ISV-VR as a safe and efficient way to construct a convenient and comfortable observation environment to replace the “field trip.” It allows students, especially those who are unable to participate in field trips, to observe the landscape more carefully, and enabling students to go where they cannot in real life.

T1: In fact, using ISV-VR in writing class is more like an electronic version of “field trip,” and it’s more efficient and safe. Actually, I can’t take a whole class of students to have field trips often. Such opportunities are very rare for students. While ISV-VR can offer students more opportunities to observe the world. So basically I think this is a “field trip in the classroom.”

T16: There are things and sights in ISV-VR that I haven’t ever seen before. So, I think this is a good opportunity for students to observe things what they don’t have the chance to see in their daily life.

T6: Using VR can save a lot of time and is more convenient than traditional field trip.

T4: Students who use VR can look more carefully without causing misunderstanding and embarrassment, because many students will be embarrassed to observe in real life in crowded places, or observe other people.

In this category, teachers are inclined to substitute the physical field trip with ISV-VR experiences. The field trip is a well-established pedagogical means for immersive learning. Nonetheless, it is a difficult teaching activity to implement due to logistical and safety considerations. Teachers in this category focus on the advantages of VR technology in circumventing the time and space limitations and in providing a safe and efficient learning environment. This conception is grounded in the primary technological affordances of VR. Description about in-depth processing of the meaning of what students observed is lacking.

#### Improving Students’ Writing Skills

In the second category, teachers believe the purpose of using ISV-VR is to improve students’ writing skills. ISV-VR served as an information-rich environment that can stimulate detailed observation. Teachers believe that by observing in ISV-VR, students can acquire more writing materials and practice various descriptive skills in writing. Besides, the rich writing materials provided by ISV-VR can stimulate students to engage in more detailed descriptions, so that the content of their composition is more detailed and vivid.

T7: I think the main use of ISV-VR is to allow students to find more details that they usually don’t notice or they can’t imagine. They can find these in ISV-VR, and make these things their writing materials so that their composition can be vivid and concrete. Besides, they can also have some new subjects to write about.

T10: When you read the students’ work, you will notice that the pictures they have in their minds are not clear, so their description is monotonous. But after using ISV-VR and with my encouragement and guidance in the classroom, students try to use more descriptive techniques in their compositions, so the content of the composition is much more specific and authentic. I think students’ writing is actually better than last semester.

T21: I notice that in the VR writing classroom, some students asked me, “Teacher, I see the sky is very vey blue, how should I write?” I told him that he could use words like ‘azure blue’ or ‘bright blue’ instead of ‘very blue.’ I think VR can help students to describe more vivid and realistic pictures by using rich words.

T8: One writing skill students need to learn is the “walking description method,” and another writing skill is the “fixed-point description method.” There are videos and static images in VR, students can practice using these two writing skills.

In this category, teachers begin to link ISV-VR observation with the content of the writing, and their focus is on the students’ writing with more things and with more varied techniques. The teachers believe that writing is a process from input to output while observing in VR can increase students’ input to writing, so as to improve their writing performance.

#### Promoting Students’ Learning Participation and Motivation

In the third category, ISV-VR is conceptualized as a vehicle to promote students’ learning participation and motivation, and a tool to change students’ attitudes toward writing. Teachers believe that the freshness brought by this cutting-edge technology attracts students to participate in learning activities more actively. Teachers also get more responses from their students, and the interaction between teachers and students is enhanced so that the learning effect is reportedly improved. Moreover, the benefits brought by ISV-VR technology to students’ writing will improve students’ confidence in writing, thus further enhance their motivation to study and write.

T11: When students observed a certain location in ISV-VR, they sometimes exclaimed: “Wow, it’s so beautiful, I want to go there on Saturday!” They talked about their feelings, because they were touched by the scenery, and I’m pleased about that. I think it’s so great to stimulate their interest in observation and writing.

T18: The writing ability of my students is relatively weak. But after using ISV-VR, some of them became serious about writing. If they haven’t finished watching ISV-VR in school, I would give them the website links of the panorama pictures, and they watched the pictures at home. I feel that students are willing to spend time and effort to prepare for their writing.

T13: When students observing in the VR, I would walk around the class, and some students would ask me a lot of questions about scenes they observed in VR, and they are more involved in the classroom.

T5: In fact, I am very surprised by the effect of the class, because my students are usually very quiet in classroom, there are 20 girls and 2 boys in my class. In the past, I kept asking them questions, but they didn’t communicate with me very well. However, in the VR classes, I hope they can answer the questions when I ask them, and they all did as I expected.

In this category, teachers generally focus on the students’ learning attitude. Teachers say they are pleased that students’ attitudes toward writing have changed. They are more concerned with the affective dimension of learning to write rather than the technical means-end aspect of writing. The students’ learning attitude and motivation are foregrounded rather than the stipulated learning outcomes to be achieved.

#### Shifting Learning From Teacher-Centric to Student-Centric

This category encapsulates the teachers’ view of the utilization of VRDW as shifting learning from teacher-centric to student-centric. In traditional writing classrooms, teachers’ role is to teach the selected articles for reading comprehension and explicate the embedded writing techniques for students to emulate. The VRDW creates a shared immersive environment where students can pursue their own perspectives while the teachers facilitated by providing personalized guidance to stimulate students to think deeper. From this perspective, the ISV-VR platform provides students with opportunities to form personalized connections with the places and people they observed. The difference between this category and other categories is that teachers are actively aware of their roles as understanding facilitators. This includes strategies for questioning and discussion to develop students’ personal thinking and understanding. This change has also increased the interaction between teachers and students.

T16: My (teaching) habit is that I don’t give students too much guidance first. I ask them to observe in the ISV-VR and then write down what they are interested in. after that, I give them some ideas, I give each of them as different advice as possible.

T14: In my ISV-VR class, my role is still a teacher, but what I teach is not content anymore. In the past, when I was teaching, I mainly started with my point of view, but now students don’t use my point of view anymore. I mainly lead them to associate what they observed in VR with their life experience, to imagine and reflect a little bit.

T17: When observing in VR, students can freely choose the viewpoint from 360 degrees, pause at any time to observe the details of the scene they are interested in, or shuttle between the scenes. Students decide the scene to observe, the order of observation, and the scene to be described.

T9: I find that in the VR class, there is no need for me to give them a topic or a scene to describe, because students decide what to write on their own, then I help them with the details of writing and guide then to think deeper.

The concept of teachers in this category shows that teachers are no longer the only source of knowledge in the classroom. They realize that the protagonist in the ISV-VR classroom has become students, and the teacher’s role has changed from lecturing to guiding. This shift is essential to open up space for students to assume the responsibility of being the author of their composition. Teaching is to promote students’ learning and understanding through cooperation with them. The cooperation includes helping, motivating, asking questions, and discussing with students. With this in mind, the teachers explored as much as possible the students’ various understandings and ideas and expand these differences. VR triggered diverse responses from students and consequently more personalized responses from the teachers. This contributes to a more student-centric learning rather than the more monologic teaching that is based solely on reading passages.

#### Enhancing Collaborative Learning Among Students

The differences in what the students experienced in the ISV-VR environment, both as individuals and as groups, can be shared and compared in classroom discussion sessions and this has provided diverse perspectives for rich discussions and learning to occur. Based on the differences, some teachers conceived VRDW as a pedagogical environment to promote collaborative learning. This is achieved through having a shared 3D environment that affords different and individual angles of observation that triggered different experiences. Collaboration in VRDW includes learning from each other’s perspectives via listening and commenting, which led to more refined ideas.

T15: I want students to share what they had seen in ISV-VR, and to work together on the worksheet. Then appraise and select the best work from their group. In this process, students finally came up with very good answers, and they can learn a lot from each other.

T13: I want the students to learn how to observe from the perspectives of multi-angle and multi-sensory. So I divided four groups and asked each group to focus on a specific sensory perspective to observe. I found that if the students focused on a certain angle, they could observe more deeply, and that also create points for peer discussion. At last, when the groups reported their results, every group can learn from each other.

T2: I rarely organized collaborative learning in traditional writing classes in the past. However, VR offers students a learning environment where they can observe collaboratively. Students can choose their point of view 360 degree in the VR scene, or they can pause to observe carefully. So I found that the details observed by students are very different, so I encouraged students to observe collaboratively to enrich their findings in VR.

T18: Using VR can enhance the sharing of experiences among students. If some students have had some personal experience, other students will listen to this student’s sharing after watching VR. For example, a classmate shared his experience of shopping at a street stall: “I once bought a vacuum cleaner at a similar street stall. It was originally sold for 120 yuan. I said it had no power cord and asked the shopkeeper to sell it for 20 yuan. The shopkeeper even agreed!” After hearing this anecdote, other students felt more real about the chaotic of the street market.

In this category, in addition to being facilitators, the teachers use ISV-VR to build collaborative learning environments. The teachers intentionally select angles of observation and create points for discussion. They required the students to work in groups to summarize the observational findings and finished the learning tasks collectively. For teachers in this category, the ISV-VR is viewed as a means to engender social collaboration for deeper meaning-making afforded by diverse observational experiences.

#### Cultivating Students’ Positive Values and Moral Character

The sixth category of cultivating students’ positive values and moral character refers to teachers leading students to fully and profoundly understand the cultural and educational connotation of literary works. This conception drives the teachers to focus on building emphatic understanding between reading and writing through the embodied experience in ISV-VR that contextualizes the guided reading of the selected texts. Teachers chose these passages based on the educational connotations and the cultural content they contained, including appeals to cultural significance, positive values, and moral character through reflections on the real world. More than literary achievement and skills development, teachers pay more attention to the cultivation of students’ empathy to understand the emotions of other authors and their perception of cultural connotations, so as to increase students’ relatedness to the community.

The pedagogical activities help students to have a deeper understanding of the educational connotations and the cultural content that the authors delivered. In this process, the teachers employ the VRDW to facilitate students’ associative thinking and their ability to think from multiple perspectives, especially from the perspectives of the authors. The selected authors in turn provide the experiential framing that is culturally valued. The VR materials invoke experiential references for students to interact and understand the authors’ perspectives.

T17: It is mainly for students to understand that people have different perspectives of life and different understandings of things, and show students how other authors write about Shatin (an area of Hong Kong), so as to cultivate students’ thinking of understanding the world from multiple perspectives. It is difficult for students to get rid of their preconceived ideas, but the combination of VR and reading text will make it easier for students to develop empathy and understand the connotation of education and humanistic emotions in literary works.

T12: Finally, in fact, the most important thing is cultivating students’ positive values and attitudes, and to understand the deep meaning of the reading passage, that is, the author’s perspective expressed through the text. So the first step is for students to understand other author’s ideas and how they view the author’s ideas. It is more about emotional and moral education.

T19: For example, this is written by a boy in my class. After watching VR, he imagines that he is a cat in an old neighborhood. He describes the changes in the neighborhood where he lives in the tone of a cat, and laments the fast-paced changes in the city and the strong contrast between the slow-paced life in the old neighborhood.

T12: For example, in the article “Father and Son” describes the Wanyi Reservoir. It writes that the author went to the Wanyi Reservoir with his father when he was a child, and then he took his son with him to the same place when he grown up. Authors expressed his affections of family, through text analysis and observation of Wangi Reservoir in VR, students can experience the feelings described by the author in the text and thus to cultivate students’ gratitude to their loved ones.

In this category, the texts and observations in ISV-VR are described as sources of knowledge or ideas, and students became active meaning creators by citing authoritative sources with social value. In selecting passages for reading comprehension, teachers chose articles that reflect deep and critical issues that shape the author’s choice of what to describe the places based on the themes established in each unit (e.g., the disjoint between new and old communities, the gap between rich and poor, the diversity of the city life). Students think more deeply under the guidance of the teacher and produce new knowledge. In this process, the students’ thinking is also developed and improved.

#### Shaping Students’ Self-Identity as “Writers”

Based on the previous categories, in this category, teachers led students to compare and contrast the literary works with reference to the scenes that students observed in ISV-VR, so as to enable students to further develop their unique views of the landscapes, people and communities. In other words, detailed observation of the scenes and studying how the scenes are depicted by established writers create deep understanding about how objective scenes are subjectively transformed into meaningful sociocultural artifacts that can engage readers in a unique and emotive time-space created by the authors. The teachers aim to develop students’ voices in their writing through reading and they led the students to think about their own relationship with others and communities. Students then write about their unique experiences, opinions, and affections with reference to the viewed VR landscapes. This conception seems closely reflects Norton’s (1997) conception of language and identity depicted as “how students understand their relationship to the world” (Norton, 1997, p. 410). It is arguably the core of being a writer and the teachers intend to facilitate students to discover possible themes and to express how these ideas from both the selected passages and VR materials are connected to themselves and their understanding of the world.

T13: So I think what’s going on in ISV-VR is that students are learning how to observe like a writer, and to associate with what’s going on. Yes, the connection between observation and association, which is equally important, and it’s actually the hardest thing to teach. But only by associating with their own life experiences can students form their own unique perspectives.

T17: Students may have different ideas about a community, there can be criticism, also some personal outpouring. So there’s a lot of thinking. Like a lot of ideas and associations that come out of it. For example, what are people’s impressions of this area in the past? What should be my relationship with the community? These questions could inspire them to think about the relationship between people and communities and reinforce their writer identity.

T13: Before using VR, there was no way to teach students how to observe like a writer. So how to teach students to see from a writer’s point of view? It need teachers to show them how to observe, for example, a wall here is mottled, why it has become so, it turns out that these details can reflect the long history of ancient buildings. When students know how to observe like a writer, they can have new findings in daily life and their own viewpoints.

T12: Because some of the scenes in VR are the places where students live, students find that they never observed the places they passed by everyday. In fact, the ultimate goal is to enable students to take the initiative to observe in real life and realize that the whole world is closely related to them. For example, no matter what the teacher says or the text describes how the rocks on the beach were formed through the vicissitudes of time, the students have no experience about it. Only when they see the traces left by the change of the stone can they feel the changes in the vicissitudes of time. And then they want to visit on the spot, I think this is the most meaningful, because these feelings are real.

In this category, teachers realize that descriptive writing has a function of establishing personal sociocultural bonds between the student writers and the places, which transform descriptions into meaning making. Excellence in descriptive writing necessarily reflects meaningful space, time and communal relationships (Hyland, 2002). Students study the Hong Kong landscape literature and observe the corresponding landscapes and living environment via ISV-VR. The sensitization of the context is reinforced through the reading of related passages and receiving teachers’ guidance. The concurrent and convenient access to the scene was for the teachers an enriched and supportive environment for them to cultivate students’ writer identity. The teachers thus nurture students’ connection to local culture and community. Teachers in this category emphasizes students’ role as writer and facilitate students’ development of individual meaning making, which gives rise to students’ sense of identity as writers.

### Relationships Among Categories

The relationship among categories and how the categories are arranged are reflected in a defined structure (Marton and Pong, 2005). By comparing and contrasting the similarities and differences between categories, three key aspects of the relationships emerged from the data analysis: (1) conception’s orientation, (2) teaching attention locus, and (3) understanding of writing. As the category level rises, the complexity and inclusiveness of experiencing VRDW will also increase. Åkerlind et al. (2005) pointed out that the hierarchical structure is not based on value judgments, but represents that certain categories include other categories and are therefore more complicated than others. Table 2 shows the key aspects that illustrate the relationship between the categories defined in this study.

**TABLE 2:** Key aspects of experiencing VRDW.

Key aspects	Category 1	Category 2	Category 3	Category 4	Category 5	Category 6	Category 7
	Offering students more observational opportunities	Improving students’ writing skills	Promoting students’ learning participation and motivation	Transforming learning from teacher-centric to student-centric	Enhancing collaborative learning among students	Cultivating students’ positive values and moral character	Shaping students’ self-identity as “writers”
Conception’s orientation	Skill-oriented	Skill-oriented	Community-oriented	Community-oriented	Community-oriented	Identity-oriented	Identity-oriented
Teaching attention locus	Content	Content	Meaning/concept	Meaning/concept	Meaning/concept	Culture	Culture
Understanding of writing	Cognitive activity	Cognitive activity	Social activity	Social activity	Social activity	Cultural activity	Cultural activity

The “conception’s orientation” refers to the teachers’ underlying understanding of VRDW. The first two categories mark the teachers’ conceptions as “skill-oriented.” The teachers’ purpose for the application of ISV-VR in writing instruction is to improve students’ writing skills, implicitly referencing performances measured by examination. The orientation of conceptions in the 3, 4, and 5 categories shifts to “community.” Teachers believe that ISV-VR technology can provide a social learning environment for students. This shift positioned the VRDW to include socialization as part of the process of writing, which is arguably a richer conception of writing. The orientation of conception ultimately shifts to creating a socio-cultural “writer identity” in the latter two categories, as teachers believe the use of ISV-VR technology can help students develop their voice in writing. This shift emphasizes the subjectivity of writing through personal meaning making, which builds a unique writer identity.

As to the second aspect, teaching attention locus, the teachers’ attend to three areas of teaching that is hierarchical. In categories 1 and 2, teachers instruct their students to focus on quantity of the “content,” which enables students to make observations and enrich their composition by using more descriptive writing skills. In categories 3, 4, and 5, teachers focus on promoting students’ engagement and collaboration that ISV-VR provides. The teaching attention was on creating 21st century learning experiences (Chai et al., 2019). In the category 6 and 7, teachers focus on the ultimate aim of writing curriculum: cultivating writer who can construct unique and socioculturally meaningful essays. They seek to cultivate the students’ understanding and arouse their interest in local culture and care for their communities. This works by allowing them to observe local representative landscapes and read literary works depicting these landscapes.

The third aspect is the teachers’ understanding of writing. In the 1 and 2 categories of teachers’ conception of teaching descriptive writing with ISV-VR, teachers regard descriptive writing as a cognitive activity. Descriptive writing is viewed as product-oriented or text-focused. At this point, they consider ISV-VR as a tool to enrich the content of students’ compositions. In the 3, 4, and 5 categories, teachers consider descriptive writing to be a social activity. Apart from individual learning, teachers emphasized more on the interactional aspect of writing classes. In the 6 and 7 categories, teachers consider descriptive writing to be a cultural activity. Students learned literary works with cultural significance, enhanced their understanding of local culture through observations in VR, and expressed their ideas of local culture in writing. Students can acknowledge involvement in a particular community through descriptive writing (Hyland, 2002). Moving across the categories in this hierarchy also marks a shift from focusing on teachers to focusing on students, and from emphasizing products to emphasizing processes. Emphasizing products means that teachers’ learning focuses on analysis of students’ performance, execution, success or final results, which can be measured by indicators such as the use of rhetoric and descriptive writing skills. Emphasizing products means that teachers’ learning focuses on the orchestration, dynamics and deployment of the students’ mental process, and other variables that modulate the acquisition of observation abilities (García-Martín and García-Sánchez, 2018).

### Distribution of Categories

Teachers’ individual experiences may distribute across categories. Therefore, the frequencies of each teacher’s conceptions are tabulated in Table 3 to gain a better understanding of how teachers’ conceptions of VRDW spread across the different categories. The frequency was measured according to how many times the participants stated a certain idea. To see the characteristics of each teacher’s conceptions more intuitively, the frequencies were further converted into symbols in Table 4. If a teacher’s conception is identified to a category, then this category will be marked with a “√,” and the most frequently mentioned categories were marked with a “▲.” For example, in Table 3, the VRDW conceptions for T5 are distributed in categories 1 to 6, where category 2 is the most frequently mentioned category (18 counts). Therefore, categories 1 to 6 of T5 are marked with “√,” and Category 2 is marked with “▲.”

**TABLE 3:** Frequency distribution of teachers’ conceptions of VRDW.

	Category 1	Category 2	Category 3	Category 4	Category 5	Category 6	Category 7
T1	2	4	4	6	0	1	0
T2	1	3	4	1	2	5	1
T3	6	2	2	3	2	2	1
T4	2	2	7	4	0	0	0
T5	2	1	6	2	3	1	0
T6	2	3	7	1	3	0	0
T7	0	4	8	12	0	0	0
T8	0	4	10	0	0	0	0
T9	4	6	2	0	0	0	0
T10	1	4	8	3	1	1	0
T11	0	4	12	0	1	0	0
T12	3	0	5	2	0	20	17
T13	2	11	7	6	5	12	3
T14	1	1	7	3	2	1	0
T15	1	4	6	3	2	1	0
T16	5	2	6	8	0	5	0
T17	0	1	1	7	5	3	2
T18	0	3	6	9	0	0	0
T19	2	3	6	1	2	8	0
T20	3	3	4	7	0	1	0
T21	1	5	8	0	3	0	0

**TABLE 4:** Frequency distribution of teachers’ conceptions of VRDW.

	Category 1	Category 2	Category 3	Category 4	Category 5	Category 6	Category 7
T1	√	√	√		√▲	√	
T2	√	√	√	√	√	√▲	√
T3	√▲	√	√	√	√	√	√
T4	√	√▲	√		√		
T5	√	√▲	√	√	√	√	
T6	√	√▲	√	√	√		
T7		√	√		√▲		
T8		√▲	√				
T9	√	√	√▲				
T10	√	√▲	√	√	√	√	
T11		√▲	√	√			
T12	√	√			√	√▲	√
T13	√	√	√	√	√	√▲	√
T14	√	√▲	√	√	√	√	
T15	√	√▲	√	√	√	√	
T16	√	√	√		√▲	√	
T17		√	√	√	√▲	√	√
T18		√	√		√▲		
T19	√	√	√	√	√	√▲	
T20	√	√	√		√▲	√	
T21	√	√▲	√	√			
	√ = 16	√ = 21	√ = 20	√ = 12	√ = 17	√ = 13	√ = 5
	▲ = 1	▲ = 9	▲ = 1	▲ = 0	▲ = 6	▲ = 4	▲ = 0

As can be seen in Table 4, improve students’ writing skills (Category 2) appeared in every teacher’s conceptions (*n* = 21) follow by promoting students learning participation and motivation (Category 3, *n* = 20), the frequency of shaping students’ self-identity as “writers” (Category 7) is the least (*n* = 5). Besides, the highest frequency of the teachers’ mentioned category was improving students’ writing skills (Category 2, *n* = 9) followed by transforming learning from teacher-centric to student-centric (Category 4, *n* = 6). The majority of teachers’ most sophisticated conceptions of VRDW is cultivating students’ positive values and moral character (Category 6, *n* = 8).

## Discussion

This study aims to explore the variations of teachers’ teaching conceptions in the context of VRDW. The results offer an understanding of how language teachers assimilate and accommodate new technology in writing education by revealing specifically different ways that teachers experienced VRDW. Teachers’ experience of adopting innovation in education is a concern among educators (Alshahrani and Al-Shehri, 2012; Newton and Beverton, 2012; Hsieh and Tsai, 2017; Liu et al., 2017). In this study, seven conception categories described along with teacher interview excerpts formed a spectrum for understanding the teachers’ conceptions. The structural relationship and hierarchy further explained the internal connection between the conceptions. Finally, the distribution of the categories presented a better understanding of the spread of the teachers’ individual experiences of VRDW.

In the context of language learning, VR technology has been advocated for a long time. However, it has been pointed out that few studies explored from the perspective of teachers (Lin and Lan, 2015). Studies also revealed limitations in teachers’ conceptions of ICT-supported language learning (Alshahrani and Al-Shehri, 2012; Newton and Beverton, 2012; Liu et al., 2017). It advocates that we need more nuanced studies on teachers’ conceptions of involving VR technology in writing education. Studying teachers’ conceptions of VRDW enables educators to gain a sense of how teachers think about teaching goals, activities, strategies, tasks, and processes in this context. In addition, examining teachers’ conceptions can also provide insight for teacher trainers and policymakers to promote teachers’ professional development. The results of this study may provide insight for better integrating new technology (especially ISV-VR) into the school curriculum.

Consistent with previous research, the more commonly reported conceptions are similar to the surface conceptions found in this study. In category 1 and category 2, teachers considered ISV-VR as enriching writing material and improving students’ writing skills. From the aspect of the conception’s orientation, the surface conceptions found in the current research were skill-oriented. They are similar to the orientation of surface conceptions that Wang and Matsumura (2019) found: writing was the application of skills and strategies. The teaching aim was to enable students to demonstrate their ability to understand and apply specific reading and writing skills. In terms of teaching attention locus, the surface conceptions which were found in the current research focus on the content rather than the meaning and concept. Students’ observation in ISV-VR was to enrich their composition. Similarly, Wang and Matsumura (2019) pointed out that literary works were regarded as objects of practice writing, rather than sources of knowledge and ideas. From the aspect of teachers’ understanding of writing, teachers who held the surface conceptions in the current research regarded writing as a cognitive activity. In the same way, Boulton-Lewis et al. (2001) founded that the superficial conceptions of teaching were considered to impart information or skills, and teachers and content were the focus. These surface conceptions focus more on the technology and means-end aspects of writing, taking ISV-VR acting as a tool to achieve a better writing performance. Such conception may be limiting in that it may result in students viewing and experiencing the writing curriculum as merely a school work to be completed.

On the other hand, the more sophisticated conception categories focus more on deepening students’ understanding of the world and improving students’ thinking. From the perspective of the conception’s orientation, the more sophisticated conceptions summarized in the current research were community-oriented. In category 3, the teacher believed that the application of ISV-VR can promote students’ learning participation and motivation. Similarly, Lin (2015) pointed out that teachers were applying ICT to let students become “more focused” and “more engaged” in the writing class. In category 4, teachers regarded VRDW as transforming learning from teacher-centric to student-centric. It is also consistent with the sophisticated conception that Wang and Matsumura (2019) noted: teachers tend to support students in exploring writing topics rather than direct instruction. It also shares the same orientation with the sophisticated conception of “transformation” that Boulton-Lewis et al. (2001) summarized: the teacher organized the situation to provide the stimulus for students to take action, and then faded into the background. In terms of the teachers’ understanding of writing, in sophisticated conception categories, teachers considered writing as a social activity. The fifth category is enhancing collaborative learning among students. Lin (2015) also found that teachers considered the application of ICT as engaging students in discussion and increasing interactions between teachers and their students. From the perspective of the teaching attention locus, in the more sophisticated conceptions in this study, teachers focused more on meaning and concept. It is similar to the sophisticated conception that Wang and Matsumura (2019) found: reading and writing were integrated through emphasizing a deeper understanding of the text. This process led the students to a “learning through thinking” model (Lin, 2015) which rely much on social negotiation that makes the thinking visible. Teachers possessing such conceptions can create enriching 21st century learning experiences with technology but it may at times miss the key purpose of developing writers.

Finally, we found that some teachers with strong sophisticated conceptions would connect writing with culture, aiming to develop students’ writer identities. This finding sheds new light on our understanding of teachers’ conceptions in writing education. Teachers who held the last two categories of conception believed that VRDW was an approach to reinforce students’ self-identities as “writers.” Although developing students’ writer identities is a great challenge (Freedman and Ball, 2004; Vasudevan et al., 2010), the teachers apparently exploit the immersive situational learning environment to stimulate students’ inner voice supplemented by literary works from established writers and teacher’s guidance. Teachers with sophisticated conceptions would guide students in developing empathy about other people’s emotions and help inspire students’ inner voices. Therefore, teachers’ sophisticated conceptions shape how they configure the learning environment for students’ writing development. These teachers would work together with students to establish their personal meaning by stimulating students’ unique opinions and feelings about the communities and landscapes when students experiencing communities and landscapes closely in immersive VR learning environments. Bower and Jong (2020) reckoned that much of the success of IVR learning depends on the pedagogical approaches that occur outside the IVR environment. Teachers who hold sophisticated conception leverage the pedagogical affordances of VR by incorporating guided reading of literature and embodied experience in the VR learning environment. The coordination between text and sensory experience allows teachers with this conception to pose questions and offer guidance about text interpretation with multi-dimensional observation. This help students to develop refined understanding about the aspects of words, rhetoric, structure, and narration. These teachers also employ traditional means such as using worksheets for students to record the details their findings of close reading. The teachers encouraged students to think from multiple perspectives and deepen their understanding and feelings of communities. Our findings appear to be well supported by the arguments held by writing educators. From the research on the literature of writing education, we find the basis that supports teacher’s conception of applying VR to develop student’s writer identity. As suggested by Vasudevan et al. (2010), students could draw on their cultural resources to establish “literate identities” in the classroom by employing multimodal digital tools for composing. Identity creation involves a self-conscious selection from the varied cultural resources at one’s disposal. This characterization is also consistent with Bakhtin’s theory on how people understand and incorporate the voices of others. Namely, different cultural resources offer different roles (Freedman and Ball, 2004). From the aspect of the conception’s orientation, this conception is identity-oriented. Teachers who held this conception focused on the cultural context and regarded writing as a cultural activity. This result not only shows the high level of teachers’ conceptions of writing and VRDW but also increases our knowledge of helping students constructing their writer identities. The proposed structural relationships of the categories provide insights into the nature and variations of teachers’ conceptions of VRDW. Meanwhile, they also help to have a more complete understanding of teachers’ writing conceptions. From the distribution of individual experiences of teachers, it was found that only few teachers had such sophisticated conceptions, and most teachers didn’t realize the role of VR in cultivating students’ cultural consciousness and writer identities. This may lie in the fact that the teachers’ conceptions of VRDW are closely related to their conceptions of writing, and the teachers’ conceptions of writing may not reach higher levels. Our study suggests that in future studies, researchers may need to develop teachers’ sophisticated conception in the professional development program to better promote the integration of technology into school curriculum (Hsieh and Tsai, 2017).

We are aware that our research may have two limitations. First, the leaders of the participated school in this study are enthusiastic about integrating ICT into the curriculum, and they have rich attainments in writing education. This may affect teachers’ conceptions toward VRDW. The conceptions obtained in this study may be more positive than that of other schools. Therefore, more schools need to be involved in the follow-up research to examine the variations in teachers’ conceptions of VRDW in a different school environment. Second, the results of this study mainly come from the interview data of teachers. Therefore, we need to pay attention that there may be a certain inconsistency between the teachers’ discourse and their teaching practice. However, we do not try to generalize the results of this study. Instead, readers of this study need to decide to what extent they transfer the results of this article to their own context. Also, the teachers’ conceptions obtained in this study can be applied as the basis for formulating the assessment criteria of teachers’ attitudes toward ISV-VR, or even other technology-supported writing learning, or as a framework for formulating the curriculum of language teacher education.

## Conclusion

The current study investigated variations of Chinese teachers’ conceptions of VRDW. It revealed seven conception categories, the distribution of the categories in teachers’ conceptions, and the hierarchical relationships of the categories. The findings of this study have some important implications for teacher educators, policymakers, and school administrators in schools, especially for those who are concerned with VR-supported language learning. This research not only corroborates previous results about teachers’ conceptions of writing education but also expands our understanding of teachers’ experience of applying VR technology in L1 writing. From the perspective of teacher education, the results of this study can provide references for language teachers’ professional development to promote VR-based immersion language learning in the school curriculum. If teacher educators want to make VR devices play more of their potential in writing education, it is necessary to cultivate more sophisticated conceptions of VRDW among teachers. Applying the results of this study, teachers can reflect on their teaching practice of adopting VR technology to promote students’ learning of writing in their native language, to expand and enhance their understanding of immersive writing learning supported by VR.

It is also necessary to realize that teaching practice may vary depending on the teacher’s educational background and environment. Therefore, future research can explore the conceptions of VRDW held by teachers in different educational environments, so as to understand teachers’ conceptions and implementation of VRDW more comprehensively. Moreover, future research can explore the consistency of teachers’ teaching implementation and their conceptions in this context, to find and explain how the conceptions affect their implementation. Also, the conceptions of VRDW held by students also need to be explored in future research, to find out the connections and inconsistency of teachers’ and students’ conceptions, so as to find and solve the potential gap.

## Data Availability Statement

The original contributions presented in the study are included in the article/supplementary material, further inquiries can be directed to the corresponding author.

## Ethics Statement

The studies involving human participants were reviewed and approved by Local Ethics Committee of the Chinese University of Hong Kong. The participants provided their written informed consent to participate in this study.

## Author Contributions

MC and C-SC contributed to the conceptualization, research design, data collection, and analysis. MJo contributed to the project’s principal investigator, funding acquisition, research and development of the ISV-VR system, and overseeing the project. MC, C-SC, MJo, and MJi contributed to the manuscript writing. All authors approved the submitted version.

## Conflict of Interest

The authors declare that the research was conducted in the absence of any commercial or financial relationships that could be construed as a potential conflict of interest.
